# EEG-vigilance regulation is associated with and predicts ketamine response in major depressive disorder

**DOI:** 10.1038/s41398-024-02761-x

**Published:** 2024-01-26

**Authors:** Cheng-Teng Ip, Mateo de Bardeci, Golo Kronenberg, Lars Hageman Pinborg, Erich Seifritz, Martin Brunovsky, Sebastian Olbrich

**Affiliations:** 1 Center for Cognitive and Brain Sciences, University of Macau, Taipa, Macau SAR China; 2 Neurobiology Research Unit, University Hospital Rigshospitalet, Copenhagen, Denmark; 3 Hospital for Psychiatry, Psychotherapy and Psychosomatic; University Zurich, Zurich, Switzerland; 4 Epilepsy Clinic, University Hospital Rigshospitalet, Copenhagen, Denmark; 5 National Institute of Mental Health, Klecany, Czech Republic; 6 Charles University, Third Faculty of Medicine, Prague, Czech Republic

**Keywords:** Predictive markers, Physiology, Psychiatric disorders

## Abstract

Ketamine offers promising new therapeutic options for difficult-to-treat depression. The efficacy of treatment response, including ketamine, has been intricately linked to EEG measures of vigilance. This research investigated the interplay between intravenous ketamine and alterations in brain arousal, quantified through EEG vigilance assessments in two distinct cohorts of depressed patients (original dataset: *n* = 24; testing dataset: *n* = 24). Clinical response was defined as a decrease from baseline of >33% on the Montgomery–Åsberg Depression Rating Scale (MADRS) 24 h after infusion. EEG recordings were obtained pre-, start-, end- and 24 h post- infusion, and the resting EEG was automatically scored using the Vigilance Algorithm Leipzig (VIGALL). Relative to placebo (sodium chloride 0.9%), ketamine increased the amount of low-vigilance stage B1 at end-infusion. This increase in B1 was positively related to serum concentrations of ketamine, but not to norketamine, and was independent of clinical response. In contrast, treatment responders showed a distinct EEG pattern characterized by a decrease in high-vigilance stage A1 and an increase in low-vigilance B2/3, regardless of whether placebo or ketamine had been given. Furthermore, pretreatment EEG differed between responders and non-responders with responders showing a higher percentage of stage A1 (53% vs. 21%). The logistic regression fitted on the percent of A1 stages was able to predict treatment outcomes in the testing dataset with an area under the ROC curve of 0.7. Ketamine affects EEG vigilance in a distinct pattern observed only in responders. Consequently, the percentage of pretreatment stage A1 shows significant potential as a predictive biomarker of treatment response.

**Clinical Trials Registration:** https://www.clinicaltrialsregister.eu/ctr-search/trial/2013-000952-17/CZ

**Registration number:** EudraCT Number: 2013-000952-17.

## Introduction

Depression is a debilitating mental disorder that can be resistant to conventional treatments such as serotonin and noradrenaline reuptake inhibitors (SNRIs) and selective serotonin reuptake inhibitors (SSRIs) or to psychotherapy. Recently, ketamine has emerged as a promising intervention for therapy-resistant depressive disorder [1], due to its rapid onset of action and effectiveness in patients who have not responded adequately to conventional antidepressant agents. Although various formulations of ketamine exist, the intravenous route of application has been shown to result in higher response rates compared to intranasal esketamine [2].

However, ketamine is not guaranteed to result in a response or remission, and ~50% of patients show no response after a single infusion [3]. There is a need for a more stratified psychiatry [4] to identify patients who are mostly likely to benefit from a ketamine intervention. Previous markers have been identified as potential predictive markers for ketamine response, including high body mass index (BMI) [5], depressive symptoms and previous suicide attempts [5], early onset of depression, chronic and treatment-resistant course of the disorder [6], family history of alcohol dependence [7, 8], genomic variants [9] and the dissociative side effects during and post-infusion, which up to now yield mixed results [10, 11]. Furthermore, functional magnetic resonance (fMRI) imaging has yielded evidence for decreased connectivity between several regions involving the anterior cingulate cortex (ACC) and prefrontal cortex (PFC) [12] or amygdala [13] as predictors of response. However, others find increases in connectivity associated with a favorable treatment outcome following ketamine interventions [14, 15].

Direct electrophysiological measures such as electroencephalogram (EEG) offer a non-invasive and cost-effective way to directly measure brain function when compared to other sophisticated device-based biomarkers, e.g., fMRI or positron emission tomography (PET). Especially the concept of EEG-vigilance, a term was coined a century ago by British neurologist Sir Henry Head [16]. It reflects the quantification of EEG-wakefulness patterns as a decline of functional brain states from full wakefulness and alertness after closing the eyes toward more sleepy and drowsy brain states just before sleep onset. The concept of EEG vigilance regulation dates to physiologists like Head, Loomis, Bente and Roth [16–19] and was later adapted and advanced into an electrophysiological framework for affective disorders [20]. Notably, an algorithm has been developed [21], validated [21–23] and replicated [24, 25]. This algorithm considers the spatial distribution of EEG power spectra and the whole framework addresses the changes over time of these spectra. Thus, the algorithm enables the categorization of 1 s EEG epoch into distinct vigilance stages, including stages 0 (indicating the highest level of vigilance regulation), A1, A2/3, B1, B23, and sleep stage C (representing sleep onset, see supplementary materials S.5). Most individuals naturally undergo transitions between these different vigilance stages as part of their physiologic vigilance regulation. However, some individuals remain within high vigilance stage, a condition referred to as “hyperrigid” or “hyperstable” vigilance regulation. Conversely, others exhibit rapid declines into lower vigilance stage, signifying unstable vigilance regulation [20–22]. Earlier findings involving healthy subjects have demonstrated a shift of EEG spectra toward slow and fast oscillations (i.e., delta and gamma activities) following ketamine administration [19, 26–28], resembling the “dissociation of vigilance” [19], while results for schizophrenia patients have been inconsistent [27, 28]. This decrease of cortical vigilance, even with subanesthetic dosages, is a well-established effect of the compound [26] and was reported only a few years after the first synthetization of ketamine.

Depression manifests as disruptions in vigilance regulation, often causing patients to experience heightened arousal and difficulty falling asleep [29–31]. These alterations are accompanied by various physiological changes, such as increased sympathetic tone [32, 33], elevated activity in the hypothalamic–pituitary–adrenal (HPA) axis [34, 35] and decreased REM sleep latency [36, 37]. In line with clinical observation, the EEG-vigilance framework [20, 21, 38] posits that depression is associated with ‘hyperrigid’ or ‘hyperstable’ EEG vigilance regulation during rest. Specifically, this pattern involves heightened occipito-parietal alpha activity (termed as A stages in EEG vigilance regulation) and reduced theta/delta activity (which would be B and C stages) in patients with depression [39]. Previous studies have shown that responders to fluoxetine exhibit greater pretreatment occipital absolute alpha power when compared to non-responders and healthy controls [40]. Moreover, lower relative delta and theta powers have been associated with better treatment response to paroxetine [41]. The EEG vigilance algorithm has demonstrated excellent diagnostic and predictive capabilities in major depressive disorder (MDD), with a fast transition to lower vigilance stages has been found to predict a favorable response to SSRI [24], a result that has been recently replicated in a large, independent cohort using a 3 min resting EEG [25]. In addition, research has found an association between baseline EEG activity and the response to ketamine in MDD. Lower baseline gamma activity and larger post-infusion gamma power associated with better antidepressant response [42].

To clarify these associations and identify potential markers of response, this study aimed to analyze the effects of intravenous ketamine on EEG vigilance in patients with depression and to examine whether high pretreatment EEG vigilance, characterized by heightened alpha and low delta/theta activities, could serve as a predictor for the response to ketamine treatment. Data from a placebo-controlled, single-blind, one-arm, fixed sequence design without randomization trial of intravenous ketamine infusion in patients with MDD were analyzed retrospectively. We test the generalizability of pretreatment EEG vigilance by evaluating its performance on an independent testing dataset. It was hypothesized that ketamine breaks a hyperrigid vigilance regulation and decreases resting state vigilance after application. Ketamine was further hypothesized to be more effective in patients that showed EEG wakefulness patterns related to high vigilance.

## Materials and methods

### Subjects

**Fig. 1:**
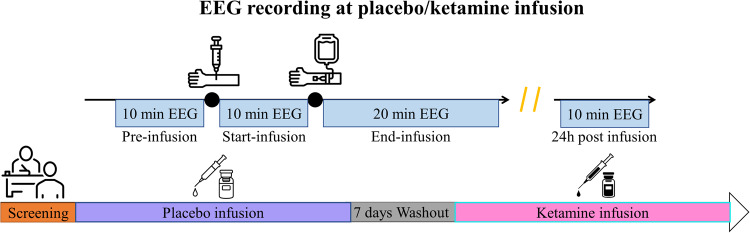
Overall study design. Each patient was allocated to a placebo infusion followed by a ketamine infusion with a 7-day washout period. Resting EEG was recorded at pre-, start-, end- and 24 h post-infusion.

The full study details have been previously reported [43, 44]. In brief, 24 patients suffering from MDD (recurrent or single episode, age 18–65 years) were recruited (EudraCT Number: 2013-000952-17) between 2010 and 2015 (see S.1 in the supplementary materials for the flow diagram of the study). All participants received a placebo infusion followed by a ketamine infusion after an interval of 7 days (see Fig. 1 for the study design). All participants provided written informed consent prior to inclusion. The study aimed to identify predictors of response to a single intravenous dose of ketamine as a treatment for depression (monotherapy or combination) in patients with MDD. Inclusion and exclusion criteria were diagnosis according to DSM-IV criteria confirmed using the Mini-International Neuropsychiatric Interview—M.I.N.I., Czech version 5.0.0 [45]. Further main inclusion criteria were Score ≥ 20 on the Montgomery-Åsberg Depression Rating Scale (MADRS), ≥1 prior non-response to adequate antidepressant treatment in the current major depressive episode (while in total 21 of the participants could be classified as TRD with ≥2 antidepressant trials during the current episode), and being on a stable dose of drugs for depressions for a minimum of four weeks prior to admission. Treatment augmentation by lamotrigine, lithium, antipsychotics and monoamine oxidase inhibitors was not allowed. Exclusion criteria included any suicidal risk assessed by clinical examination, current psychiatric comorbidity on Axis I and II, serious unstable medical illness or neurological disorder.

Testing dataset (*n* = 27) is coming from the same research site and is registered at clinicaltrialsregister.eu (EudraCT Number: 2009-010625-39). It is a double-blind, placebo controlled, randomized, crossover study evaluating the psychotomimetic effects of a single ketamine infusion in patients with MDD. Details of this study have been previously described [43, 44]. Identical ketamine infusion regime, MADRS treatment response (>33% at 24 h after infusion from baseline), inclusion and exclusion criteria were applied in the original and testing dataset.

The study was approved by the Ethical Committee of Prague Psychiatric Centre/National Institute of Mental Health, Czech Republic and was performed in accordance with the ethical standards of the Declaration of Helsinki 1975, revised Hong Kong 1989.

### Clinical measures and treatment response

Severity of depressive symptoms was assessed at baseline (i.e., before infusion), and 24 h, 72 h and 7 days post infusion using the MADRS. Response to treatment was defined as a decrease of depressive symptoms by means of MADRS > 33% at 24 h after infusion from baseline. This modified response criterion was used to include more patients who showed a fast-acting response to ketamine within 24 h but did not meet the 50% criterion. The same criterion was used in a previous study on the same cohort [44].

### Ketamine infusion

A unilateral intravenous catheter was inserted into the subjects’ forearm for ketamine infusion. Racemic ketamine hydrochloride (Calypsol, Gedeon Richter Plc., Czech Republic) was administered using an infusion pump (ID 20/50, Polymed medical CZ Ltd). Ketamine was dispensed in a loading dose of 0.27 mg/kg for the first 10 min (start-infusion), followed by an infusion of 0.27 mg/kg within 20 min (end-infusion, Fig. 1). Thus, the total dose was 0.54 mg/kg within 30 min. More details on the infusion regime can be found elsewhere [44]. Ketamine and norketamine blood levels were assessed via blood samples respectively 10 min and 30 min after the first loading dose of ketamine.

### EEG recordings

Electroencephalogram (EEG) recordings were acquired by a BrainScope digital amplifier (M&I, Prague, Czech Republic) with the subjects sitting in a semi-recumbent position, eyes closed in a sound-attenuated room with subdued lighting. EEG acquisitions for both the original dataset and the testing dataset were conducted in a similar manner [44]. EEG was recorded from 22 channels with an extended international 10-20 system (Fp1, Fp2, F3, F4, C3, C4, P3, P4, O1, O2, F7, F8, T3, T4, T5, T6, Fz, Cz, Pz, AFz, A1, A2) and additional horizontal electrooculogram (HEOG) channels were placed at the outer canthi of each eye. The data sampling rate was 1000 Hz for the original dataset and 250 Hz for the testing dataset. Resting EEG was recorded 10 min before and 30 min during infusion. Another 10 min resting EEG was recorded 24 h post infusion to investigate any remaining drug effects (Fig. 1). Impedances were kept below 50 kΩ.

### EEG processing and classification of EEG-vigilance

Twenty-five VIGALL EEG channels were selected and or interpolated from the EEG net according to the VIGALL manual (VIGALL 2.1 manual; https://research.uni-leipzig.de/vigall/). The EEG/EOG data of the original dataset were down sampled to 250 Hz to match the sampling frequency of the testing dataset. Subsequently, the data were re-referenced to an average reference and further processed in Brain Vision Analyzer 2.0 (Brain Products GmbH, Glitching, Germany). Artifact segments were manually marked but not removed to retain the full vigilance-time-series for each subject. The percentage of removed segments across all conditions remained below 13%. No significant differences were observed in the percentage of removed segments across different conditions (see supplementary materials S.3.1 and S.3.2). Filters (bandpass: 0.5/70 Hz; 50 Hz notch) were applied after correcting eye-movement artifacts, cardioballistic artifacts and technical disturbances (independent component analysis approach). No significant differences were observed in the number of excluded ICAs across different conditions (see supplementary materials S.4.1 and S.4.2). EOG channels were bandpass filtered (0.01/70 Hz) to retain slow eye movements. All data were included in the further analysis.

Slow eye movements (SEMs) criteria were set to 100 µV with a 6-second window length to detect any drowsiness in the recording [46, 47]. Each 1 s epoch was automatically classified into the following arousal states using the algorithm-based Vigilance Algorithm Leipzig (VIGALL 2.0), resulting in a vigilance time-course: stage 0 (highest arousal), A1, A2, A3, B1, B2/3, C (lowest arousal, sleep onset, classified visually by sleep grapho-elements from an experienced rater). In the current analysis, as well as in prior studie [24, 25, 48], the prevalence of stages A2 and A3 has been relatively low. Therefore, we adopted the conventional practice of combining the two A stages (A2/3) [24, 25, 48]. Since no subject showed stage C segments after visual inspection, the preprocessing resulted in five different vigilance stages (0, A1, A2/3, B1, B2/3). The VIGALL stages were assigned numerically with a range from 6 (stage 0) to 2 (stage B2/3). Percentages of the different vigilance stages and median vigilance were calculated for each 1 min-block (in a total of 30 blocks). The vigilance slope was calculated using linear regression of the median vigilance during each 1 min-block for each participant. The vigilance slopes were reported for pre-, start- and end-infusions.

### Generalizability of the biomarker on the testing dataset [43, 44]

Only patients received ketamine with pre-infusion resting EEG data were included (*n* = 24). Pre-infusion resting EEG data were processed and VIGALL classified using the approach described above. Predictive biomarker found on the original study was validated in the testing dataset. The testing dataset was used to estimate the prediction performance of the logistic regression fitted on the original dataset.

### Statistics

Repeated measures analyses of variance (ANOVA) were performed separately for three VIGALL outcomes, including median vigilance, median slope of vigilance time series and percentages of vigilance stages. Separate ANOVA was performed for each vigilance stage. In addition to examining the complete resting-state recording, VIGALL outcomes of first 3 min-block for each timepoint (pre-, start-, end-infusion) were also analyzed following previous predictive findings [24, 25]. Moreover, VIGALL measures the dynamic fluctuation of different functional brain states which are sensitive to the length of recording. Therefore, the first dose (i.e., the loading dose; start-infusion) and the second infusion (end-infusion) were considered as two separate treatment conditions. Only the first 10 min of the end-infusion was included in the analysis. Age was included as a covariate in all models, following the previous studies [24, 25]. Bonferroni’s correction was used for multiple comparisons and post hoc analyses. Degrees of freedom were corrected by Greenhouse-Geisser correction when necessary. The significance level was set to *p* < 0.05 for median vigilance and medial slope and was set to *p* < 0.01 to control for type I error when examining the percentages of the five vigilance stages. Group differences in sex, age were tested by *χ*^*2*^ statistic and pretreatment 24 h MADRS scores were tested by one-way *F* statistic. The detailed statistics were divided into the following three parts to address the primary hypotheses:

### Ketamine effects in patients with MDD

The ketamine effects on vigilance regulation were assessed by comparing data from both ketamine and placebo infusion conditions. To reduce the number of comparisons in the models, the relative changes of vigilance were defined by subtractions between start- and end-infusion from pre-infusion before ketamine/placebo administration. Therefore, intervention (ketamine vs. placebo), treatment conditions (start-pre vs. end-pre) and the recording block (first 3 min-block/first 10 min-block) were included as within-subject factors. Group (responders vs. non-responders) was included as between-subject factor. Further partial correlation analysis was performed to assess the relation between VIGALL measures and ketamine levels in serum (both ketamine and ketamine metabolite norketamine). Specifically, ketamine blood levels 10 min after injection were used to correlate with start-pre condition and ketamine blood levels 30 min after injection was used to correlate with end-pre condition.

### VIGALL parameters as predictive biomarkers for treatment

To investigate the predictive value of VIGALL measures, only pre-infusion data were pooled from both ketamine and placebo infusions. Intervention (ketamine vs. placebo) and the recording block (3 min-block/10 min-block) were included as within-subject factors. Group (responders vs. non-responders) was included as a between-subject factor. We then validate the candidate VIGALL biomarker on the testing data by submitting the recording block (3 min-block/10 min-block) and group (responders vs. non-responders) to repeated ANOVA. Meta-analysis was performed using Revman 5.3 to calculate the mean difference and 95% confident intervals (CI). The original study and the testing study were tested for heterogeneity by the *χ*^*2*^ and *I*^*2*^ statistics.

#### Estimation of prediction scores

For each patient, the percentage of A1 stage prior to the ketamine infusion from both the original dataset and testing dataset was used as a feature to predict treatment response. We fitted a logistic regression model without regularization using the percentage of A1 stage of the 3-min block and 10-minute block exclusively of the original dataset. Only the best-performing model on the original dataset was subsequently used on the test set. From the testing set, we derived the ROC curve as well as accuracy, F1 score, precision, and recall. The odds ratio was calculated in the testing set using the optimal classification threshold of the original dataset.

### Remaining CNS ketamine effects in patients with MDD 24 h after the first dose

The VIGALL outcomes at pre-infusion and 24 h post infusion were included to examine the remaining CNS ketamine effects. Similarly, the relative changes (day2-pre) were computed before subjected to further analysis. Intervention (ketamine vs. placebo) and the recording block (3 min-block/10 min-block) were included as within-subject factors. Group (responders vs. non-responders) was included as between-subject factor.

## Results

### Sociodemographic

The sociodemographic and clinical variables are presented in Table 1 for the original data. Since there was only one responder to the placebo intervention, the between-group comparisons were only performed for the ketamine intervention. Responders to ketamine had a significantly lower MADRS score 24 h after intervention (*F*(1,22) = 9.80, *p* = 0.005) and a greater improvement on the MADRS relative to non-responders (*F*(1,22) = 10.63, *p* = 0.004, Table 1). No significant differences were found for sex, age and the pretreatment MADRS score (*p* values > 0.65). The demographics variables for the testing dataset is presented in the supplement materials (S.2).

**Table 1:** Sociodemographic characteristics for placebo and ketamine interventions.

	Placebo		Ketamine	
Clinical outcome 24 h after infusion (n)	Responders (1)^a^	Non-responders (23)	Responders (12)^a^	Non-responders (12)
Sex (M/F)	0/1	7/16	3/9	4/8
Age (Mean ± SD)	56	43.1 ± 12.5	42.6 ± 14.7	44.8 ± 10.3
Pretreatment MADRS	38	26.8 ± 4.2	27.6 ± 5.8	27.1 ± 3.9
24 h MADRS	19	27.4 ± 4.9	17.4 ± 7.5^b^	25.1 ± 4.0
ΔMADRS (% changes from 24 h to pretreatment)	50%	-2.5% ± 10.0%	35.1% ± 28%^b^	7.2% ± 10.0%

*MADRS* Montgomery–Åsberg Depression Rating Scale.

^a^Responders were defined by at least 30% improvement of depressive symptoms assessed by MADRS score after 24 h after interventions.

^b^Between group comparisons only performed for the ketamine intervention. The results showed that responders to ketamine had significant lower MADRS score 24 h after infusion compared to non-responders (*F*(1,22) = 9.80, *p* = 0.005); responders to ketamine had significant higher improvement on MADRS score 24 h after infusion compared to non-responders (*F*(1,22) = 10.63, *p* = 0.004). No significant was found for other demographic characteristics (*p* values > 0.65).

### Ketamine effects in patients with MDD

**Fig. 2:**
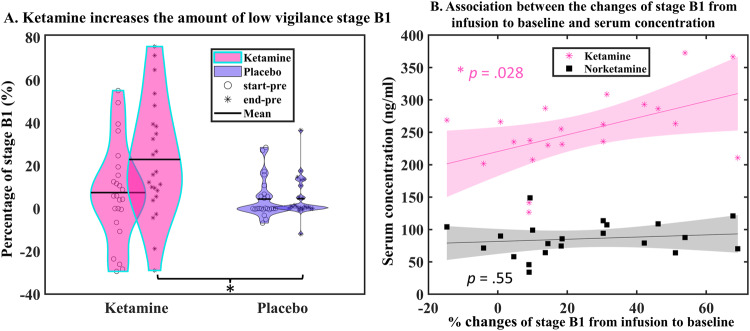
Effects of ketamine on EEG vigilance in associaton with serum levels in MDD patients. **A** Ketamine increases the amount of low vigilance stage B1. Relative to pre-infusion, ketamine increases the amount of stage B1 during end-infusion compared to placebo in all patients with depression, regardless of their clinical outcome. **B** Association between the changes of stage B1 from infusion to baseline and serum concentration. A positive partial correlation was found between ketamine serum concentration 30 min after the first loading dose and the percentage changes of stage B1 at the second infusion to pre-infusion. Note: *p < 0.05. start-pre, relative changes from the first loading dose (start-infusion) to pre-infusion; end-pre, relative changes from the second infusion (end-infusion) to pre-infusion.

**Fig. 3:**
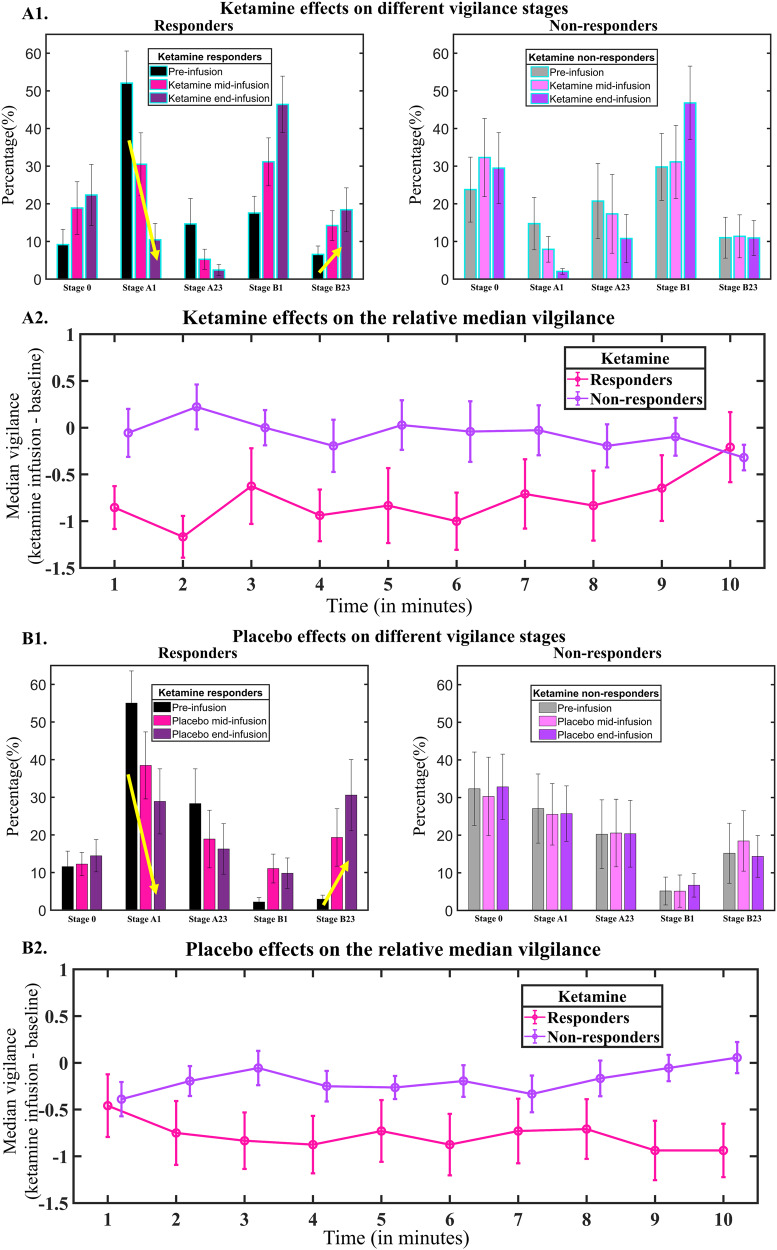
Pharmacological effects (upper panel showing ketamine effects and lower panel showing placebo effects) on different vigilance stages and relative median vigilance in MDD patients. **A1**, **B1** Responders showed a larger decrease at stage A1 and a larger increase at stage B2/3 compared to non-responders. No significant difference was found between ketamine and placebo. The mean percentage and the corresponding error bar (representing ±1 standard error) were depicted in the figure. **A2**, **B2** Mean median vigilance (averaged across start-pre and end-pre) and the corresponding error bar (represent ±1 standard error) were depicted in the figure. The median vigilance of responders decreased faster compared to non-responders. No significant difference was found between ketamine and placebo. Responders (and non-responders) were defined based on the response following ketamine infusion throughout the analysis.

We found a significant interaction between intervention (i.e., ketamine vs placebo) and treatment conditions (i.e., start-pre and end-pre) regarding stage B1 in the 10 min-block (*F*(1,21) = 8.31, *p* = 0.009). Relative to pre-infusion, post-hoc analysis revealed that ketamine increased the amount of low vigilance stage B1 during the second infusion (end-pre) compared to placebo (24% vs. 6.2%, *p* = 0.001, Fig. 2A). A positive partial correlation, corrected for age, between ketamine levels in serum and percentage changes of stage B1 from end-infusion to pre-infusion was found (*r*(17) = 0.50, *p* = 0.028, Fig. 2B) but not the percentage changes from start-infusion to pre-infusion (*p* = 0.86) nor associations between norketamine levels in serum and percentage changes from infusions to baseline (*p* values > 0.45, Fig. 2B). That is, a high ketamine concentration was associated with a greater increase in stage B1. Such an association only appeared 30 min after the first loading dose of ketamine, during end-infusion. Furthermore, the increased stage B1 effect induced by ketamine was detected in both treatment responders (*n* = 12) and treatment non-responders (*n* = 12) as indicated by the non-significant three-way interaction between intervention, conditions, and group (*F*(1,21) = 0.001, *p* = 0.98, Fig. 3A1).

Group effects were observed for median vigilance, stages A1 and B2/3. Specifically, we found significant group effects of median vigilance (3 min-block: *F*(1,21) = 4.96, *p* = 0.04; 10 min-block: *F*(1,21) = 4.90, *p* = 0.04). These results indicated that responders exhibited a faster decline in median vigilance and had higher propensities for drowsiness compared to treatment non-responders (−0.75 vs. −0.15, Fig. 3A2, B2). These effects were consistent irrespective of whether placebo or ketamine was administered (*p*s > 0.19, Fig. 3A2, B2). In addition, significant group effects were also observed for vigilance stage A1 in the 10 min-block (*F*(1,21) = 9.54, *p* = 0.006) and B2/3 *F*(1,21) = 9.26, *p* = 0.006). The analyses of simple effects demonstrated that responders had a larger decrease at stage A1 (−26% vs. −6%) and a larger increase at stage B2/3 compared to non-responders (16% vs. 2%) during the whole course of the intervention (Fig. 3A1, B1).

### VIGALL as a predictive biomarker for ketamine response and non-response

**Fig. 4:**
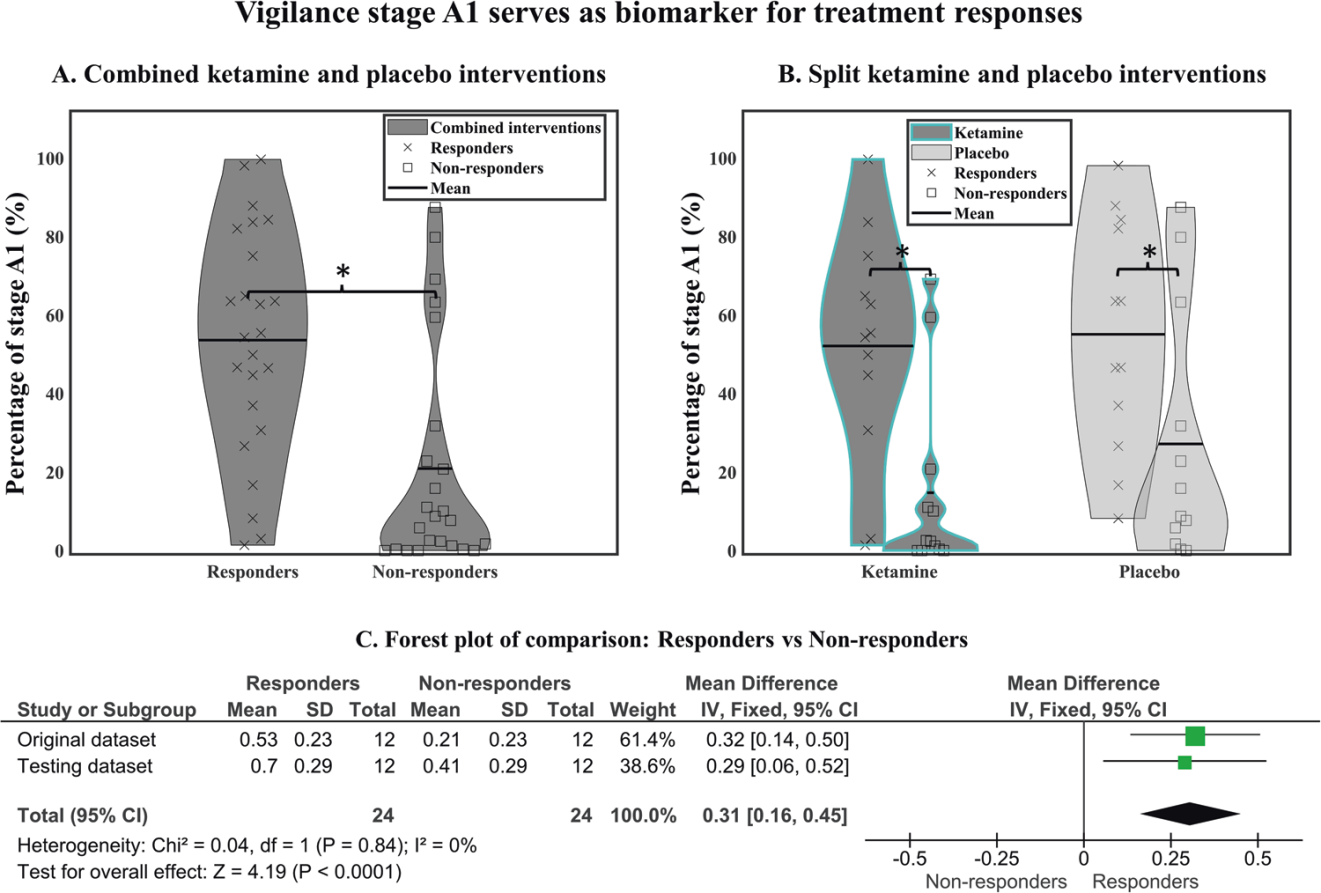
High vigilance stage A1 as an indicator of treatment responders. **A** Prior to treatment, responders had a significantly higher amount of stage A1 compared to non-responders. The effect was found in both ketamine and placebo interventions (**B**). **C** Meta-analysis of pretreatment vigilance stage A1 between treatment responders and non-responders to ketamine.

We found that, at pre-infusion, responders showed a significantly higher percentage of stage A1 compared to non-responders (3 min-block: *F*(1,21) = 8.05, *p* = 0.009, 53% vs. 23%; 10 min-block: *F*(1,21) = 11.76, *p* = 0.003, 53% vs. 21%, Fig. 4A). This pre-infusion difference for stage A1 between responders and non-responders was found in both ketamine and placebo interventions (intervention × group interaction: *ps* > .35, Fig. 4A, B), pointing toward a trait aspect in vigilance regulation for response or non-response. No significant result was found for median vigilance (*ps* > 0.25) and median slope (*ps* > 0.29).

Surprisingly, we found a significant main effect of intervention at stage B1 (3 min-block: *F*(1,21) = 8.36, *p* = 0.009; 10 min-block: *F*(1,21) = 17.05, *p* < 0.001). The baseline of stage B1 prior to ketamine infusion was significantly higher than placebo infusion (3 min-block: 24% vs. 6%; 10 min-block: 24% vs. 4%).

#### Testing dataset

In the testing dataset, high percentage of stage A1 was observed in treatment responders compared to non-responders (10 min-block: *F*(1,21) = 5.67, *p* = 0.027, 70% vs. 41%, Fig. 4C). There was a higher percentage of stage A1 observed in testing dataset compared to the original dataset (3 min-block: *F*(1, 43) = 5.48, *p* = 0.02; 10 min-block: *F*(1, 43) = 5.34, *p* = 0.03, see S.3 in the supplementary materials). However, no significant differences were found for the interaction between dataset and treatment group (3 min-block: *F*(1, 43) = 0.10, *p* = 0.75; 10 min-block: *F*(1, 43) = 0.12, *p* = 0.73). Meta-analysis revealed a significant mean difference of 0.31 (95% CI 0.16–0.45; *χ*^2^ = 0.04; df = 1 (*p* = 0.84), *I*^2^ = 0%) between treatment responders and non-responders with low heterogeneity (Fig. 4C).

#### Prediction scores

**Fig. 5:**
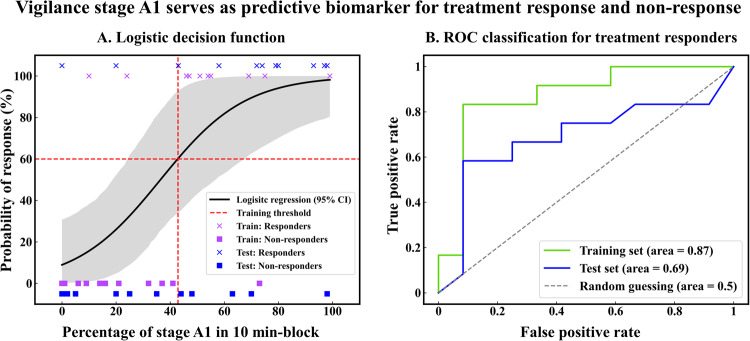
Vigilance stage A1 as a predictive biomarker in MDD. Here the training set refers to the original dataset. **A** Logistic decision functions for responders vs. non-responders. A 50% response probability represents 36% of A1 stage (only data from 10 min-block is shown here). **B** ROC classification for treatment responders derived from LOOCV.

Percentages of A1 stages were used as a predictive marker for treatment response. The 10-min block yielded the best performance in the training set (original dataset). The optimal cut-off separated the responders and non-responders with 88% accuracy (using a threshold of 43% of A1 stages, Fig. 5A). The prediction scores derived from testing data for the logistic regression yielded 67% accuracy, 0.7 f1-score, 0.75 sensitivity, 0.64 precision, and 0.7 ROC-AUC, (Fig. 5B). In summary, the odds for a patient in the testing set to be a responder are 4.2 times higher if the percentage of A1 stages is above 43% within the first 10 minutes of EEG recording.

### Remaining CNS Ketamine effects in patients with MDD 24 h after the first dose

We estimate a three-way interaction between intervention, recording block and group at stage 0 (*F*(9,189) = 2.92, *p* = 0.02 for a significant level of 0.01). No significant result was found for median vigilance (*ps* > 0.09) and median slope (*ps* > 0.27).

## Discussion

The purpose of the present study was not only analyzing changes in EEG wakefulness regulation in patients suffering from treatment resistant depression after i.v. ketamine infusion as compared to a placebo condition. Another important goal was to identify EEG-based predictors for the antidepressant effect of ketamine.

As anticipated, the results showed a clear decrease of EEG -wakefulness when comparing the pre-infusion resting state to EEG recordings after the second infusion. Especially stage B1, as a clear marker of reduced wakefulness [18, 20], increased significantly during ketamine infusion. Since this decrease in vigilance was not seen in the placebo condition, it can be regarded as a ketamine-specific effect. Further, a dose-dependency could be shown with a significant correlation between the increase of stage B1 segments and the serum concentration of ketamine. Ketamine is used for general anesthesia, where dosages of 1–6 mg and 0.4–1 mg/kg/h are required for continuous sedation [49]. Therefore, the increase in drowsiness following the two infusions with a total of 0.54 mg/kg was expected. Other studies have reported increased fast oscillations following ketamine administration [50] and some have reported increased EEG beta power during treatment with ketamine during a status epilepticus [51]. Since EEG vigilance stage B1 is defined as a low voltage EEG with prevailing EEG beta activity and loss of alpha activity [52], the findings of increased stages B1 are in line with the literature [53]. This decline of vigilance was observed in both responders and non-responders. Similar to the finding that dissociations during ketamine administration might not be related to its antidepressant effects [54], this indicates that the general anesthetic effect of ketamine seems to be independent of its antidepressant action.

Regarding differential effects of ketamine on the EEG vigilance regulation in responders and non-responders, responders showed a significantly larger decrease in high vigilance stage A1 and a significantly larger increase of low vigilance stage B2/3. It has to be stated that the response rate of 33% in this study is relatively low compared to the clinical findings, which typically report response rates of ~55% [55]. This discrepancy is due to our single administration of ketamine, whereas clinical studies often assess outcome after e.g., two weeks [55]. The general decrease of vigilance was significantly more pronounced in the ketamine condition compared to placebo. This deviation from the normal progression of vigilance stages could introduce group-level variability, potentially resulting in a less homogeneous representation of the effects of ketamine on vigilance decline. However, a higher propensity toward low vigilance stages for responders in comparison to non-responders was present in both conditions, which implies a robust predictive marker. This shows that the EEG-vigilance regulation is not a pure state but a state-modulated trait marker, as has been shown previously with the same metrics used in this study [56]. Thus, ketamine might just add to the effect of declining vigilance during rest [57]. It is important to consider preclinical research findings regarding the differential anesthetic effects and antidepressant effects of ketamine [58]. Notably, R-ketamine has shown promise as a potent, long-lasting, and safe antidepressant, with a reduced risk of psychotomimetic side effects and abuse liability when compared to S-ketamine [59]. While our study primarily focuses on vigilance decline, these findings underscore the complexity of ketamine’s effects, suggesting that its general anesthetic properties may be independent of its antidepressant actions. Furthermore, while patients with depression show a higher overall vigilance compared to healthy controls [21, 22, 25], it has been shown in large samples from the iSPOT-D trial [60] that patients suffering from MDD revealed better response to SSRIs when EEG-vigilance showed fast drops [24]. Since ketamine induces a state of decreased vigilance, it might be argued that it helps to break through a hyperrigid vigilance regulation, as it can be found in patients with MDD [21, 36, 61, 62]. Following our results, this seems not to be the case for patients with a rigid wakefulness regulation, leaving them as non-responders. If higher dosages of ketamine could help to induce lowered vigilance stages in non-responders should be a target in further investigations with a ketamine titration regime. However, the increase in ketamine dosages is limited by its cardiovascular and other side effects [63].

When only looking at the baseline EEG to identify predictive markers, responders showed a substantially higher amount of vigilance stage A1. Interestingly, this effect was also true for the baseline condition before placebo, demonstrating that vigilance regulation is a state marker [56]. It remains unclear if vigilance stage A1 could also serve as a predictive marker for other routes of administration, e.g., for intranasal [64] or oral [65] ketamine treatment. It could be valuable to see whether genetic variations, which have previously been linked to diminished anesthetic responses to ketamine in rodents [66], mighe account for the observed differences between responders and non-responders.

Further, EEG vigilance parameters showed significant differences between responders and non-responders after 24 h with responders showing less high vigilance stage 0, while compared to non-responders. This might indicate that the long-term change of wakefulness regulation induced by ketamine might be associated with the mood changes. The strong performance on the testing set of 0.7 area under the ROC curve, proves that the results can generalize beyond our particular data sample. Moreover, the observed odds ratio of 4.2 emphasizes the strong association between the EEG-vigilance marker and treatment response. Nevertheless, advanced feature engineering, exploring non-linear and ensemble models, and augmenting our dataset with new features other than EEG vigilance can achieve a more predictive model while maintaining clinical interpretability [67].

EEG dominant frequency, a critical indicator of brain activity, is strongly influenced by the complex interplay between various brain structures, particularly the thalamus and brain stem [68]. These structures play a pivotal role in modulating EEG patterns as they drive transitions between different EEG vigilance stages. The thalamus acts as a central hub in regulating the sleep-wake cycle [69] and is integral to the phase transitions observed in EEG vigilance stages [70]. Specifically, the change of wakefulness is most pronounced during transitions from stage 0 and stage A to stage B and C and vice versa. During the transition of lower vigilance stages to higher vigilance stages, the ascending reticular activation system (ARAS) within the thalamus becomes highly active, promoting wakefulness and increasing vigilance [71]. When the medial part of the brainstem’s reticular formation (FR) is activated, it triggers the ARAS, leading to a heightened state of alertness and a desynchronized EEG. This is achieved through both specific projections to relay sensory information and nonspecific projections that facilitate overall cortical activity. In addition, other brain regions, such as the hypothalamus, limbic system, and basal forebrain, contribute to the regulation of brain arousal and the dynamic changes seen during phase transitions in EEG vigilance stages [72, 73]. Ketamine, as an anesthetic agent, demonstrates a dose-dependent effect on consciousness and EEG patterns [53, 74, 75]. The contrasting effects of ketamine at low and high doses underline its versatility in clinical anesthesia. It’s worth noting that during ketamine-induced anesthesia, EEG patterns undergo significant changes, characterized by hypersynchronous delta wave bursts and fast wave activity in the neocortex and thalamus, with limited impact on limbic systems [76]. This functional dissociation in EEG activity sets ketamine apart from standard inhalational anesthetics, which tend to produce a smoother and more gradual transition from wakefulness to anesthesia. These unique features of ketamine emphasize its significance in clinical anesthesia and underscore the wide variation in the dose x EEG arousal function between ketamine and conventional anesthetics.

The EEG-vigilance stage difference between the baseline from serum and placebo condition likely stems from the non-randomized sequence. All patients received placebo first, followed by ketamine. This sequence might have caused heightened excitement during the initial placebo session, given the unfamiliar recording environment. By the second visit (ketamine administration), familiarity could have led to increased relaxation and a corresponding rise in low vigilance B1 stages. Furthermore, it should be noted that individuals at risk of suicide were excluded from our study. The final proof of an anti-suicidal effect at times when the trials were carried out was not given, it was decided to exclude this population at risk. Therefore, our findings are not to be generalized to individuals with suicidal ideation or behaviors.

Pretreatment EEG markers hold promise for improving patient stratification in ketamine treatment, potentially increasing the effectiveness of interventions—a possibility to be replicated and explored in larger-scale studies. To facilitate the complex processes of clinical certification of biomarkers used software, regulatory rules should be adapted for faster tracks toward the clinical usage. This way, all patients can benefit from the progress in the field.

### Supplementary information

Supplementary materials

## Data Availability

The dataset of the current study is available from the corresponding author upon reasonable request.
