# Supplementary material for: EEG-vigilance regulation is associated with and predicts ketamine response in major depressive disorder
Source: Transl Psychiatry. 2024 Jan 26;14:64. doi: 10.1038/s41398-024-02761-x (PMC10810879; doi:10.1038/s41398-024-02761-x)
Supplement: Supplementary file 1 — Supplementary materials [file 41398_2024_2761_MOESM1_ESM.doc]

**S.1 CONSORT 2010 Flow Diagram**

**Placebo applied**

**Ketamine applied**

**Exclusion**

**Up**

**Enrollment**

**Analysis**

Assessed for eligibility (n=27)

Excluded (n=1)

♦  Informed consent withdrawal (n= 1)

Excluded (placebo response) (n=2)

Allocated to intervention (n=26)

♦ Received allocated intervention (n=26)

Allocated to intervention (n=24)

♦ Received allocated intervention (n=24)

Analysed (n=24)
♦ Excluded from analysis (placebo response) (n=2)

**S.2 Sociodemographic characteristics for ketamine interventions for testing da**taset

|  | **Ketamine** | |
| --- | --- | --- |
| **Clinical outcome 24h after infusion (n)** | Responders (12)^1^ | Non-responders (12) |
| **Sex (M/F)** | 8/4 | 6/6 |
| **Age (Mean±SD)** | 45.0±13.1 | 40.42±11.7 |
| **Pretreatment MADRS** | 20.2±5.5 | 20.0±6.6 |
| **24h MADRS** | 8.9±6.4^2^ | 18.2±8.3 |
| **ΔMADRS**  **(% changes from 24h to pretreatment)** | 62%±29%^2^ | 8.9%±26.0% |

Notes: ^1^ Responders were defined by at least 30% improvement of depressive symptoms assessed by MADRS score after 24h after interventions.

^2^ Between group comparisons only performed for the ketamine intervention. The results showed that responders to ketamine had significant lower MADRS score 24h after infusion compared to non-responders (*F*(1,22)=8.90, *p*=.007); responders to ketamine had significant higher improvement on MADRS score 24h after infusion compared to non-responders (*F*(1,22)=22.28, *p*<.001). No significant was found for other demographic characteristics (*p* values >.38).

**Abbreviation**: MADRS, Montgomery–Åsberg Depression Rating Scale.

**S.3.1 Statistics for the number of bad epochs between conditions, treatment responses**

|  | Within-subject effects | Interaction between group and within-subject variables |
| --- | --- | --- |
| Intervention (2) | *F* (1, 22) = 0.33, *p* = 0.57 | *F* (1, 22) = 0.06, *p* = 0.82 |
| Treatment conditions (pre, start, end) | *F* (2, 44) = 2.27, *p* = 0.12 | *F* (2, 44) = 0.28, *p* = 0.74 |
| Intervention * Treatment conditions | *F* (2, 44) = 0.76, *p* = .45 | *F* (2, 44) = 1.91, p = 0.17 |
| Group (2) | *F* (1, 22) = 0.77, p = .39 | NA |

Notes: ^1^ The significance level was set at p < 0.05.

**S.3.2 Statistics for the number of bad epochs between datasets**

|  | Within-subject effects |
| --- | --- |
| Dataset (2) | *F* (1, 47) = 1.50, *p* = .23 |

Notes: ^1^ The significance level was set at p < 0.05.

**S.4.1 Statistics for the number of excluded ICAs between conditions, treatment responses**

|  | Within-subject effects | Interaction between group and within-subject variables |
| --- | --- | --- |
| Intervention (2) | *F* (1, 22) = 1.51, *p* = 0.23 | *F* (1, 22) = 0.28, *p* = 0.60 |
| Treatment conditions (pre, start, end) | *F* (2, 44) = 2.66, *p* = 0.09 | *F* (2, 44) = 0.09, *p* = 0.89 |
| Intervention * Treatment conditions | *F* (2, 44) = 2.57, *p* = 0.10 | *F* (2, 44) = 0.37, p = 0.66 |
| Group (2) | *F* (1, 22) = 0.89, p = 0.36 | NA |

Notes: ^1^ The significance level was set at p < 0.05.

**S.4.2 Statistics for the number of excluded ICAs between datasets**

|  | Within-subject effects |
| --- | --- |
| Dataset (2) | *F* (1, 47) = 0.46, *p* = 0.50 |

Notes: ^1^ The significance level was set at p < 0.05.

**S.5 Statistics for vigilance stage A1 between current dataset and testing dataset**

|  | **3 min Recording** | **10 min Recording** |
| --- | --- | --- |
| **Dataset** | *F*(1, 43)=5.48, *p*=0.02 | *F*(1, 43)=5.34, *p*=0.03 |
| **Treatment group (Responders**^1^ **vs. Non-responders)** | *F*(1, 43)=8.85, *p*=0.005 | *F*(1, 44)=13.88, *p*<.001 |
| **Dataset * Treatment group** | *F*(1, 43)=0.10, *p*=0.75 | *F*(1, 43)=0.12, *p*=0.73 |

Notes: ^1^ Responders were defined by at least 30% improvement of depressive symptoms assessed by MADRS score after 24h after interventions.

Age was included as a covariate in all models.

S.6: Description of the behavioural level (“Wakefulness”), the EEG-based “Vigilance-stages” with corresponding description and illustration of the electrophysiological patterns from EEG and EOG in the Vigilance Algorithm Zurich (“VIGAZ”) as a translation of the former algorithm VIGALL.

**
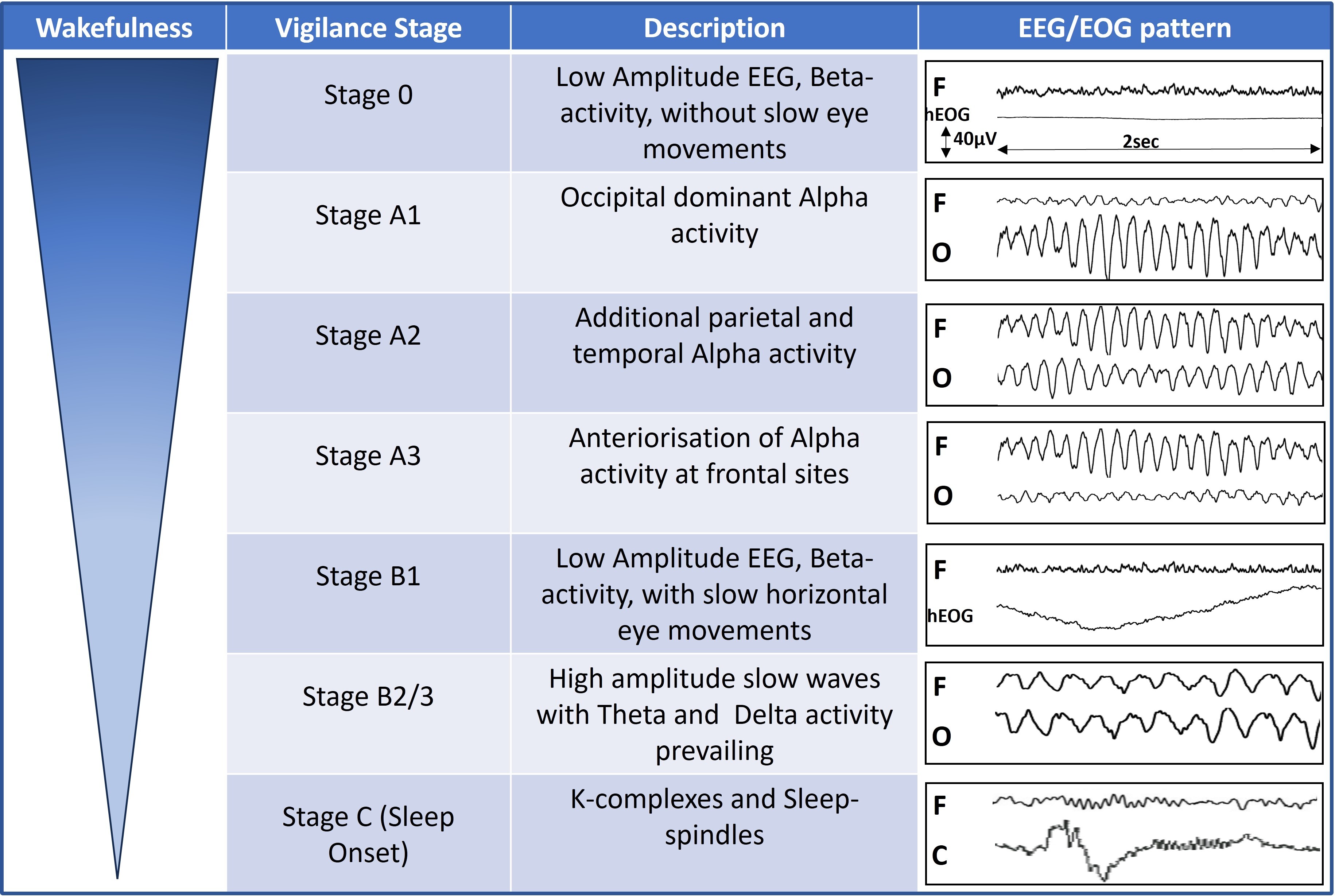
**
